# Diseases of the Gall-Bladder and Bile-Ducts

**Published:** 1898-06

**Authors:** 


**Diseases of the Gall-Bladder and Bile-Ducts.** By A. W. MAYO ROBSON. Pp. 150, 27. London: Baillière, Tindall & Cox. 1897.

In the spring of 1897, when as Hunterian Professor, Mr. Robson lectured at the College of Surgeons, we read these lectures as they appeared in the medical journals. In their present form they are enriched by numerous photographs of specimens illustrating diseases of the gall-bladder and bile ducts in the museum at the College of Surgeons and in the museums of the various London Medical Schools. Almost half the book is devoted to the consideration of the inflammatory affections of these structures; tumours are then fully treated of, and the rest of the work is taken up with the subject of gallstones. A very valuable table of a consecutive series of 170 cases of operations on the gall-bladder and bile ducts is added. Such a work as this, from the pen of a surgeon who has had a larger experience of the surgical treatment of the diseases of which it treats than any other, is of very great value indeed. Mr. Mayo Robson has had a remarkably successful series of operations for gall-stones, and a surgeon with such a record is entitled to write with authority and may well be read with advantage.

Surgical diseases of the liver, such as abscess and hydatid cysts, are not of course considered in this volume, but it covers more ground than Mr. Robson's clinical manual on gallstones. Reference is made to Mr. Robson's case of removal of malignant disease of the gall-bladder which invaded the liver. The whole mass was removed, after ligation around the liver substance below the growth, and the patient recovered. The case was reported to the Royal Medical and Chirurgical Society. We are interested to see that Mr. Robson is not in favour of cholecystenterostomy in cases of malignant growth obstructing the common duct. He thinks it open to question whether the mortality is not too high to make the operation justifiable.

Mr. Robson's attempt to explain the fact—now generally recognised—that in gallstone obstruction the gall-bladder is empty and contracted, rather than dilated, is more satisfactory than most explanations which we have seen. The so-called "ideal operation" seems to obtain more favour with Mr. Robson than it has in the past: he thinks "the serious objection to it is that the benefits of drainage are not obtained." The important subject of suppurative cholangitis is fully considered, and attention is directed to the value of drainage of the gall-bladder in such cases. The work is evidently the outcome of the labours of a busy surgeon: words are not wasted; everything is clear and concise; guides to surgical practice abound; and no surgeon will regret the time spent in its perusal. But we are bound to say that, although the book is well printed, in the presentation of his work Mr. Robson has done himself some injustice. We would rather have had an index at the end of his volume than the thirty-two pages of advertisements which are given to us, and we do not think the nine lines after his name on the title-page are in good form.

